# Recurrent Achilles Tendon Rupture in Multiple Sites as a Primary Manifestation of Systemic Lupus Erythematosus in a 32-Year-Old Patient: A Case Report

**DOI:** 10.7759/cureus.61231

**Published:** 2024-05-28

**Authors:** Miguel Jiménez-Yarza, Miguel Jiménez-Puga, Javier Ramírez-Jasso, Sergio E Vázquez-Lara, José E Sánchez-Bosque

**Affiliations:** 1 General Surgery, Instituto de Seguridad y Servicios Sociales de los Trabajadores del Estado (ISSSTE) Regional Hospital Monterrey, Monterrey, MEX; 2 Plastic and Reconstructive Surgery, Clínica Millenium, Aguascalientes, MEX; 3 Orthopaedic Surgery, Hospital Star Médica Aguascalientes, Aguascalientes, MEX

**Keywords:** ortho surgery, surgical case reports, systemic lupus erythematous (sle), achilles rupture, achilles tendon injury

## Abstract

The Achilles tendon is vital for walking and running, but it's also the most frequently ruptured tendon. Ruptures often occur without direct trauma and present with acute posterior ankle/heel pain. Various factors like age, biomechanical properties, degeneration, and mechanical factors influence susceptibility to rupture. Mechanisms of injury vary, including weight-bearing forefoot pushing off and sudden dorsiflexion of the ankle. Management goals focus on minimizing morbidity, swift recovery, and preventing complications through tailored interventions. Systemic lupus erythematosus (SLE) can also contribute to tendon rupture, especially with prolonged corticosteroid use. A 32-year-old female presented to the ER after injuring her left foot during a basketball game. She was diagnosed with an Achilles tendon rupture and underwent surgery to repair it. However, she experienced delayed wound closure and needed a skin graft. Two months later, she suffered another rupture in a different location, requiring a tendon transfer surgery. She was finally diagnosed with SLE after tests by the Rheumatology Department. Treatment commenced, and she began rehabilitation four weeks post-surgery. Surgical management of ruptured Achilles tendon involves techniques like open repair, percutaneous repair, mini-open repair, and augmentative repair. Open repair involves a direct approach with a posteromedial incision to align tendon stumps using various stitching techniques. Conservative treatment involves immobilization and non-weight-bearing for at least four weeks post surgery. For rare cases of Achilles tendon rupture caused by lupus, treatment focuses on managing the underlying disease with medications like hydroxychloroquine and glucocorticosteroids. Comprehensive evaluation, including musculoskeletal assessment, is crucial for lupus patients. SLE needs to be considered as a potential cause, especially in cases of recurrent ruptures or additional musculoskeletal symptoms. Surgical management should be tailored to individual patient needs, while also considering surgeon proficiency and preferences.

## Introduction

The Achilles tendon (AT) stands out as the thickest and strongest tendon in the human body, serving a crucial role in transmitting power from the calf to the foot, enabling fundamental activities such as walking and running [1]. The AT (or calcaneal tendon) serves as the inferior insertion point into the calcaneus for three muscle masses: the lateral and medial heads of the gastrocnemius muscle and the soleus muscle [2].

Not only is it the largest tendon but it also holds the unfortunate distinction of being the most frequently ruptured. Hippocrates is credited with the first recorded description of the injury [3], with Petit in 1726 providing comprehensive clinical and physical findings [4]. Most of these ruptures occur spontaneously, without direct trauma or skin breakage. Typically, the rupture manifests as a separation of the tendon in its body, insertion, or musculotendinous portion. The historical, symptomatic, and clinical aspects are notably specific [4], as patients often present with acute posterior ankle/heel pain, recounting a sensation akin to being kicked from behind [5].

Ruptures of the AT account for a notable 20% of all large tendon ruptures. Most of these tears occur in the watershed area, a structurally weak region located approximately 6 cm proximal to the tendon insertion in the calcaneus [1]. An incidence of 8.3 ruptures per 100,000 has been reported, peaking in the 30- to 49-year-old age group [5]. Patients can be categorized into two subgroups: young or middle-aged athletes and older nonathletes. Predominantly, AT rupture affects males, evident in a male-to-female ratio ranging from 2:1 to 12:1. Left-sided ruptures dominate. Age and tendon size play crucial roles in the biomechanical properties of the AT, influencing its ability to withstand high-stress levels. Younger individuals exhibit significantly higher tensile rupture stress and lower stiffness. Moreover, increased age, body height, cross-sectional calf muscle size, and foot size correlate significantly with thicker AT. These factors contribute to the diverse demographics and biomechanical considerations associated with AT ruptures [6].

Approximately 53% of AT ruptures occur during weight-bearing forefoot pushing off with the knee in extension, a movement commonly observed in activities like sprint starts and jumping sports such as basketball. Another 17% of ruptures occur after the sudden, unexpected dorsiflexion of the ankle, such as slipping into a hole or falling downstairs. In 10% of patients, the tendon is ruptured due to violent dorsiflexion of a plantar-flexed foot, a scenario that may unfold after falling from a height. These distinct mechanisms highlight the diverse circumstances and movements that can lead to AT injuries [3].

The precise cause of AT ruptures remains elusive, with two primary theories gaining prominence: the "degeneration theory" and the "mechanical theory." According to the degeneration theory, chronic degeneration of the tendon leads to rupture even in the absence of excessive loads. This degeneration may stem from various factors, including physiological alterations in the tendon, chronic overloading with microtrauma [6], and hypovascularity, particularly in the midsection of the tendon, observed in patients with compromised blood supply [5]. Predisposing factors encompass the use of fluoroquinolones, hormone replacement therapy, or steroids. Additionally, conditions such as ochronosis, prior AT rupture, infection, hypertension, obesity, and systemic inflammatory conditions contribute to the vulnerability of the AT [5]. Understanding these theories and associated factors is crucial for comprehending the multifaceted nature of AT ruptures.

One such inflammatory condition is systemic lupus erythematosus (SLE) a chronic autoimmune disease known for its multisystemic nature, and a course marked by periods of relapse and remission. It exhibits a notable predominance, affecting women of childbearing age at a ratio of 9:1 compared to men [7]. The incidence ranges from 0.3 to 31.5 cases per 100,000 individuals [8]. While the exact etiology is not fully understood, SLE is recognized as having a multifactorial origin. The disease presents a diverse spectrum of manifestations. In individuals diagnosed before the age of 50, cutaneous symptoms and renal abnormalities are common. Musculoskeletal involvement is prevalent, with arthralgia and arthritis affecting hands, wrists, and knees being typical manifestations. However, SLE can also present with synovitis, tenosynovitis, and myositis as the disease progresses [7]. The variability in symptoms underscores the complexity of SLE, making it crucial for healthcare professionals to consider the diverse clinical presentations when diagnosing and managing this condition.

Tendon rupture in SLE is an uncommon occurrence, predominantly affecting tendons bearing significant weight. However, a consistent factor observed in these cases, independent of SLE, is the administration of corticosteroids [9]. Spontaneous tendon ruptures tend to be more prevalent in lupus patients with prolonged disease duration, chronic corticosteroid use, and minimal disease activity [10]. This association underscores the impact of corticosteroids on tendon integrity and highlights the importance of considering these factors when evaluating tendon health in individuals with SLE.

The management goals for AT ruptures aim to achieve three primary objectives: minimizing the morbidity of the injury, optimizing a swift return to full function, and preventing complications. These objectives are typically addressed through a comprehensive approach that can be categorized into operative and non-operative interventions [6]. The chosen strategy depends on various factors, including the severity of the rupture, the patient's overall health, and individual preferences. The overarching aim is to provide effective and personalized care, ensuring a successful recovery with minimal long-term impact on function and well-being.

## Case presentation

**Figure 1:**
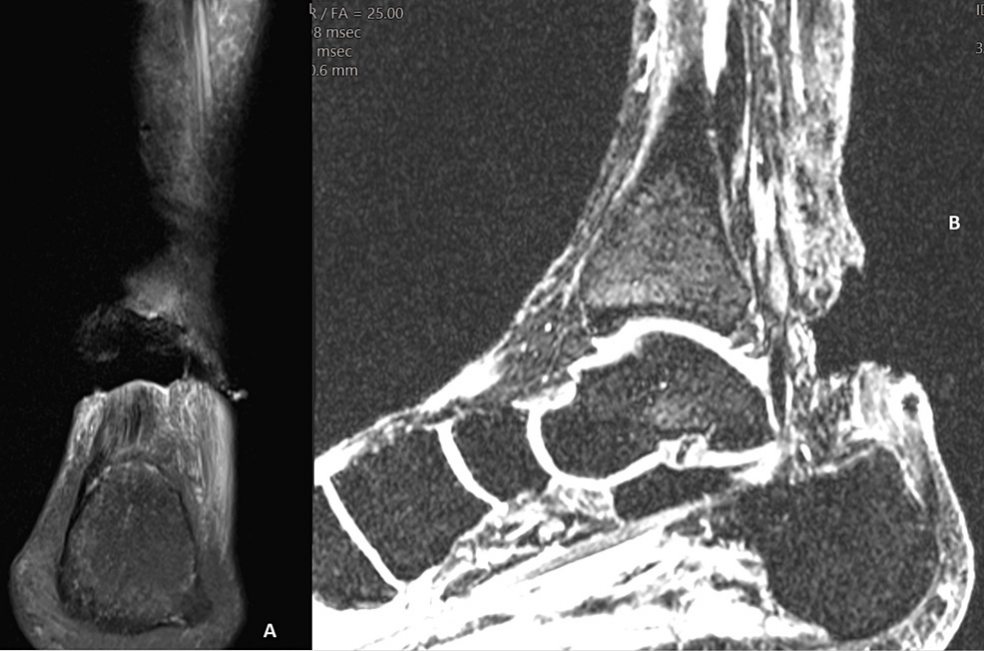
MRI images of the Achilles tendon rupture

A 32-year-old female, without apparent previous relevant medical history, presented to the emergency room with pain and noticed swelling in her left foot after a hyperextension movement in a basketball game. She also reported functional impairment of the foot. Upon consultation with the Orthopedic Surgery Department, she was diagnosed with an AT rupture. Figure 1 shows the MRI images of the AT rupture at the time of dignosis. Subsequently, she underwent surgery to repair the tendon, revealing a rupture at 2 cm from the calcaneal insertion.

**Figure 2:**
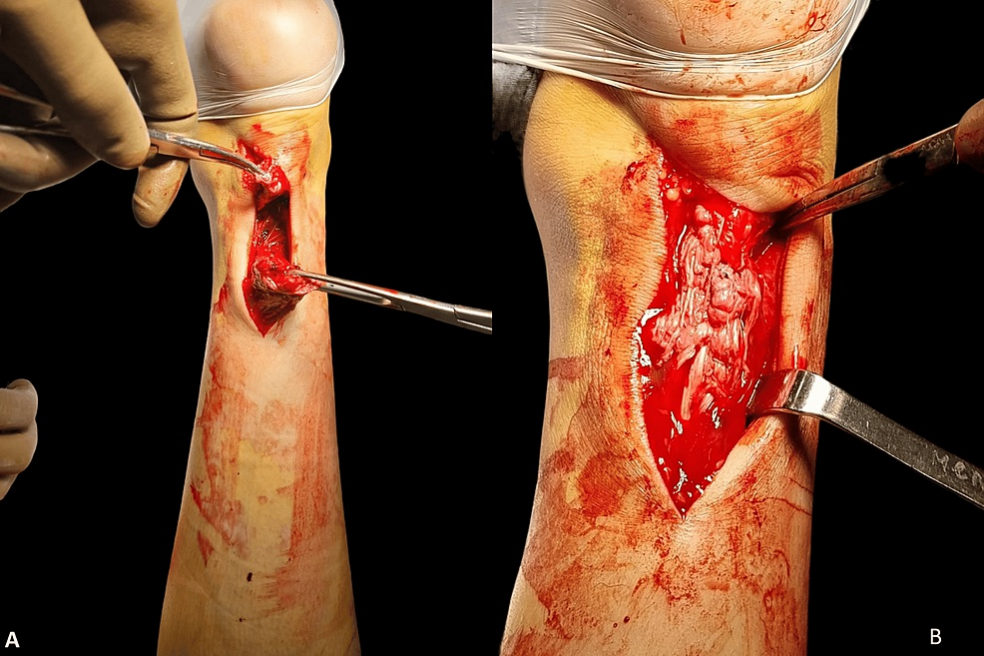
The first surgery (A) Achilles tendon rupture; (B) Repaired Achilles tendon

This was repaired using an open technique with a modified Kessler technique and anchored continuous suture reinforcement (Figure 2).

**Figure 3:**
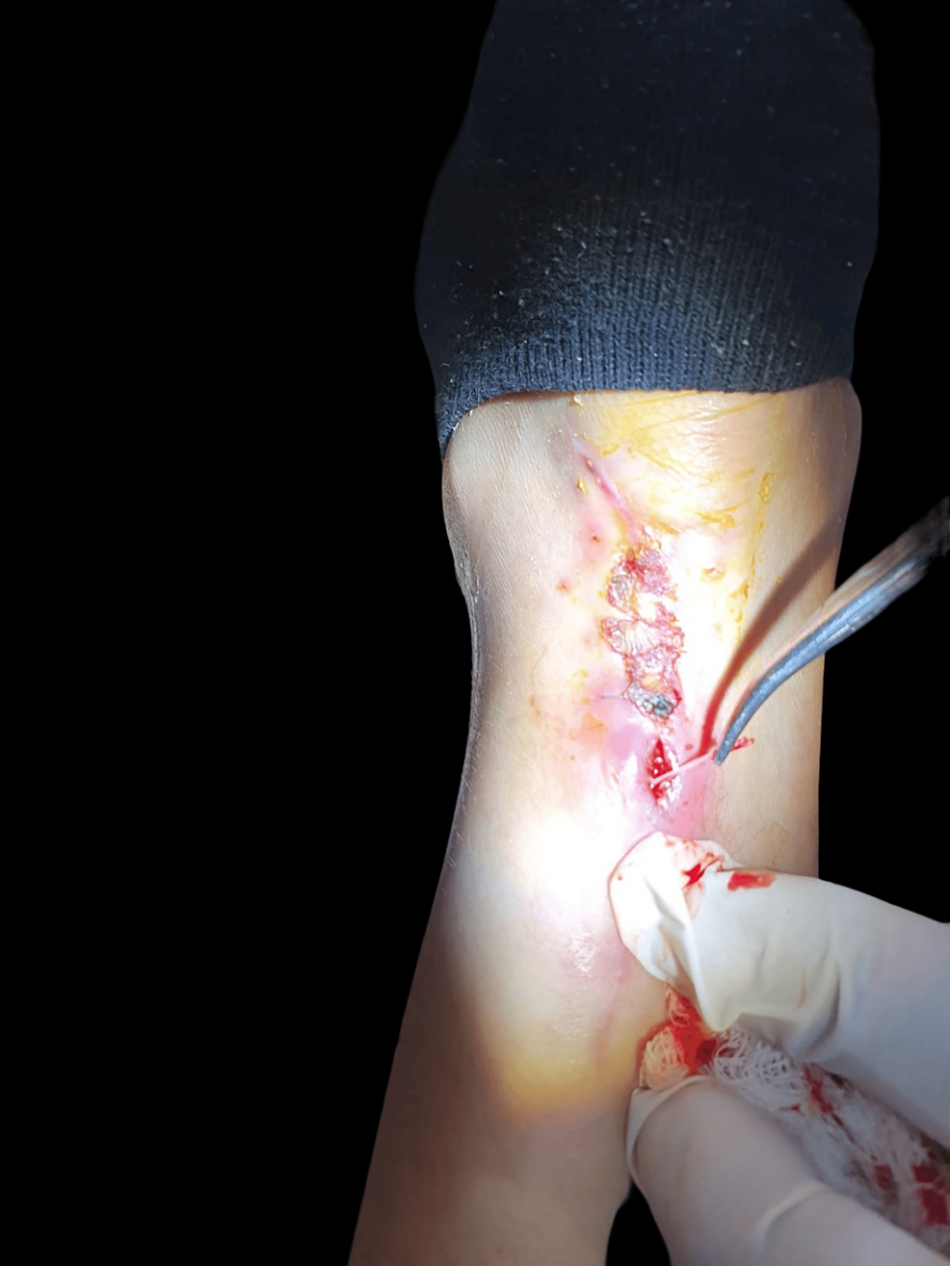
Delayed skin closure with open wound and suture exposure

Following discharge, she experienced delayed wound closure with suture exposure (Figure 3), necessitating a skin graft placement by the Plastic Surgery Department three weeks later.

**Figure 4:**
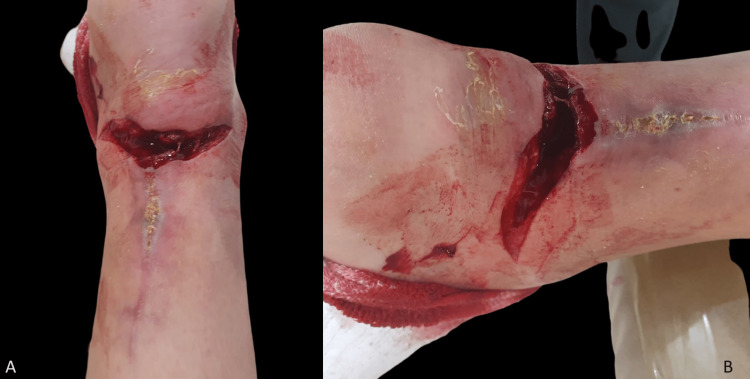
Spontaneous skin breakage on the left heel

Two months post this surgery, while following a partial non-weight-bearing treatment, she experienced pain and noticed blood on the floor as well as skin breakage (Figure 4) after placing weight on the foot.

**Figure 5:**
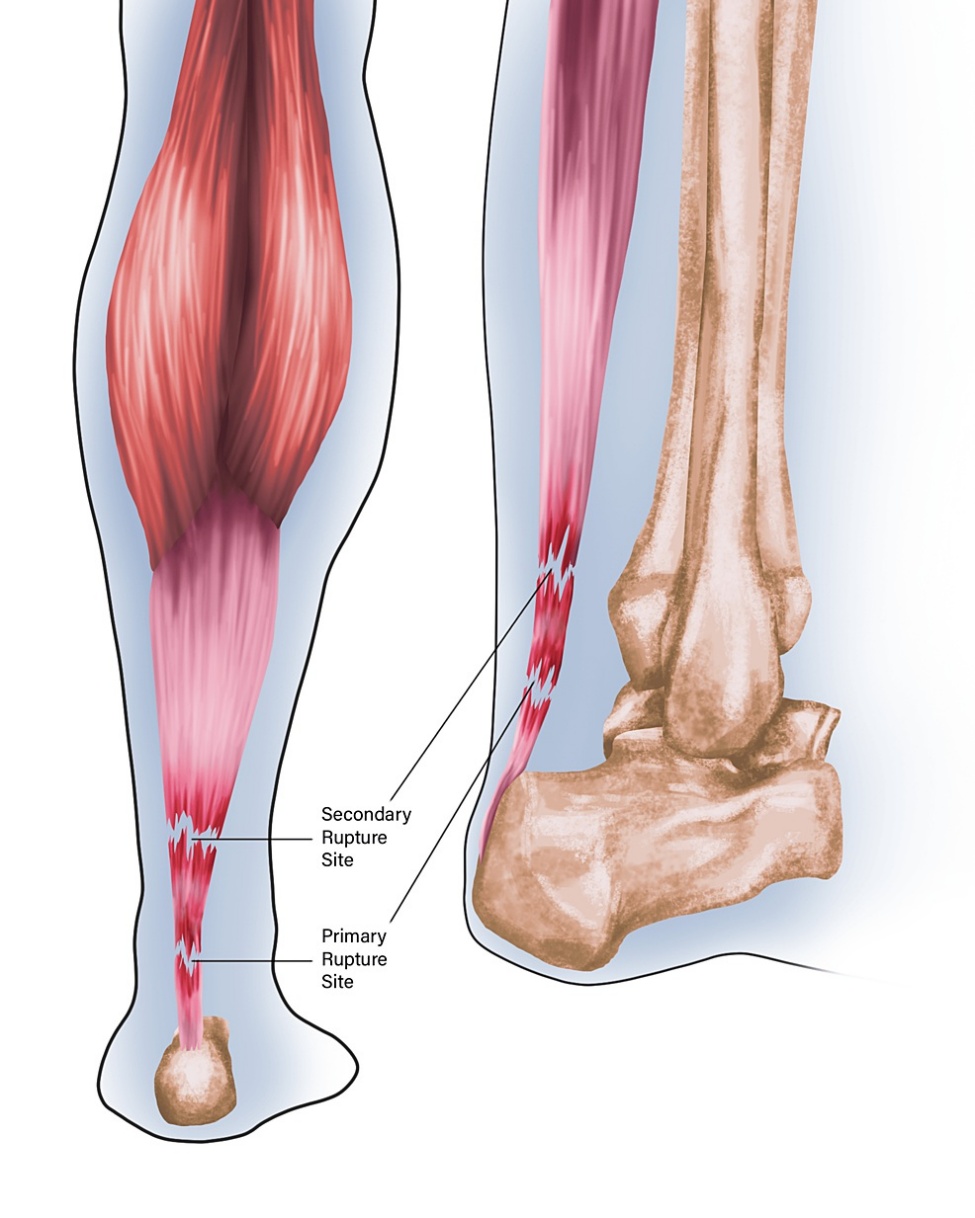
Illustration of primary and secondary Achilles tendon rupture sites Image Credits: Miguel Jiménez-Yarza

She promptly sought care at the outpatient Plastic Surgery Clinic, where a secondary spontaneous rupture of the AT, accompanied by functional impairment of the foot, was diagnosed by the Orthopedic Surgery department. Surgical exploration revealed a rupture at a different site, 7 cm from the calcaneal insertion (Figure 5), with friable tissue.

**Figure 6:**
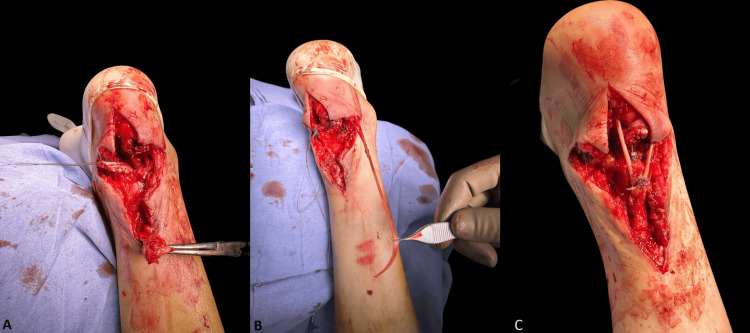
Second surgery (A) Tendon rupture; (B) Flexor hallucis longus exposed; (C) Repaired Achilles tendon with tendinous transfer

**Figure 7:**
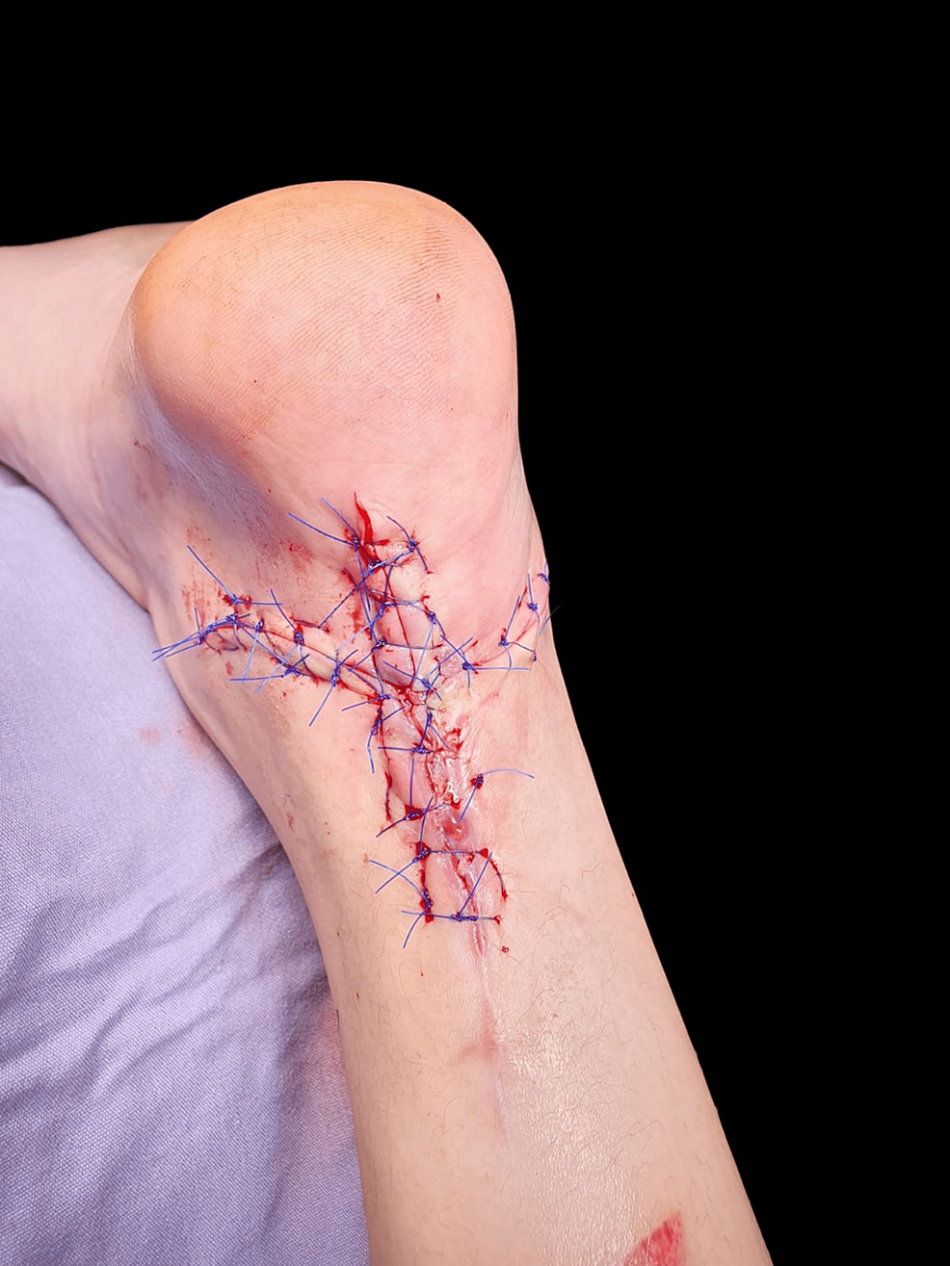
Skin suture and closure post surgery

A tendinous transfer of the flexor hallucis longus was performed to address the issue (Figure 6), followed by skin closure by the Plastic Surgery Department (Figure 7).

Due to the unusual presentation and suspicion of an underlying disease, the patient was referred to the Rheumatology Department. After conducting various tests, the patient was diagnosed with SLE. The patient commenced treatment and presented adequate skin closure, initiating rehabilitation four weeks after the surgery.

## Discussion

The surgical management of a ruptured AT involves two primary elements: the surgical technique itself and the subsequent postoperative care. Treatment options for a ruptured AT can be broadly categorized into four approaches: open repair, percutaneous repair, mini-open repair, and augmentative repair. Typically, surgical intervention is preferred for younger patients and those with higher functional demands. The direct open approach entails a straightforward procedure, utilizing an extended posteromedial incision to expose the rupture site and align the tendon stumps using various stitching techniques. It's worth noting that all types of suture materials can potentially induce local immunological and inflammatory reactions. While nonabsorbable, multifilament sutures were once favored by many surgeons, they have been associated with chronic inflammation, contamination, and infection. Nonbraided and absorbable sutures are now favored due to their adequate strength and holding capacity [11].

Conservative treatment involves immobilization and non-weight-bearing for a minimum of four weeks post surgery. Historically, non-surgical treatment has been offered to older patients with reduced functional demands or those with specific contraindications to surgery. Although rare, an AT rupture caused by lupus requires management focused on treating the underlying disease. This typically involves a combination of medications such as hydroxychloroquine, glucocorticosteroids, immunosuppressive drugs, and biological agents [12].

It's crucial to consider that the rupture may result from either the disease itself or the medication used in treatment. Therefore, when dealing with a known case of lupus or a recent diagnosis, a comprehensive evaluation should be conducted, including an assessment of the musculoskeletal system.

## Conclusions

While SLE is an infrequent factor contributing to AT rupture, it is imperative to consistently consider it as a potential cause. This becomes particularly crucial in cases involving recurrent ruptures or additional musculoskeletal symptoms, especially in individuals who deviate from the conventional demographic associated with such injuries. Dismissing the possibility of an underlying or primary cause for these ruptures is unwarranted, emphasizing the necessity for comprehensive patient evaluations. The approach to surgical management should be customized based on the specific needs and characteristics of each patient. However, it is equally important to consider the surgeon's preferences and proficiency in executing various surgical approaches.

## References

[1] Khanzada Z, Rethnam U, Widdowson D, Mirza A (2011). Bilateral spontaneous non-traumatic rupture of the Achilles tendon: a case report. J Med Case Rep.

[2] Rouviere H (2005). Human Anatomy, Vol. 3. Masson.

[3] Longo UG, Ronga M, Maffulli N (2009). Acute ruptures of the achilles tendon. Sports Med Arthrosc Rev.

[4] Reveno PM, Kittleson AC (1969). Spontaneous Achilles' tendon rupture. Radiology.

[5] Weatherall JM, Mroczek K, Tejwani N (2010). Acute achilles tendon ruptures. Orthopedics.

[6] Movin T, Ryberg A, McBride DJ, Maffulli N (2005). Acute rupture of the Achilles tendon. Foot Ankle Clin.

[7] Ameer MA, Chaudhry H, Mushtaq J (2022). An overview of systemic lupus erythematosus (SLE) pathogenesis, classification, and management. Cureus.

[8] Fanouriakis A, Tziolos N, Bertsias G, Boumpas DT (2021). Update οn the diagnosis and management of systemic lupus erythematosus. Ann Rheum Dis.

[9] Potasman I, Bassan HM (1984). Multiple tendon rupture in systemic lupus erythematosus: case report and review of the literature. Ann Rheum Dis.

[10] Chiou YM, Lan JL, Hsieh TY, Chen YH, Chen DY (2005). Spontaneous Achilles tendon rupture in a patient with systemic lupus erythematosus due to ischemic necrosis after methyl prednisolone pulse therapy. Lupus.

[11] Yang X, Meng H, Quan Q, Peng J, Lu S, Wang A (2018). Management of acute Achilles tendon ruptures: a review. Bone Joint Res.

[12] Basta F, Fasola F, Triantafyllias K, Schwarting A (2020). Systemic lupus erythematosus (SLE) therapy: the old and the new. Rheumatol Ther.

